# Texture and color enhancement imaging improves the visibility of gastric neoplasms: clinical trial with image catalogue assessment using conventional and newly developed endoscopes

**DOI:** 10.1186/s12876-023-03030-9

**Published:** 2023-11-13

**Authors:** Toshiki Futakuchi, Akira Dobashi, Hideka Horiuchi, Hiroto Furuhashi, Hiroaki Matsui, Yuko Hara, Masakuni Kobayashi, Shingo Ono, Naoto Tamai, Kazutaka Gomisawa, Takashi Yamauchi, Machi Suka, Kazuki Sumiyama

**Affiliations:** 1 Department of Endoscopy, The Jikei University School of Medicine, 3-25-8, Nishi-shimbashi, Minato-ku, Tokyo, 105-8461 Japan; 2 Department of Pathology, The Jikei University School of Medicine, Tokyo, Japan; 3 Department of Public Health and Environmental Medicine, The Jikei University School of Medicine, Tokyo, Japan

**Keywords:** Gastric neoplasms, Image enhanced endoscopy, Texture and color enhancement imaging

## Abstract

**Background:**

Texture and color enhancement imaging (TXI) enhances the changes in endoscopic features caused by gastric neoplasms, such as redness/whiteness and elevation/depression. This study aimed to demonstrate the effectiveness of TXI in improving the visibility of gastric neoplasms compared with white light imaging (WLI) using conventional (CE) and newly developed endoscopes (NE).

**Methods:**

We recruited patients who were histologically diagnosed with gastric neoplasms; endoscopy was performed, and gastric neoplasms photographed using three imaging modalities, including WLI, TXI mode 1 (TXI-1) and TXI mode 2 (TXI-2). Two different endoscopes (CE and NE) were used for the same patients. Six endoscopists provided the visibility scale scores ranging from 1 (poor) to 4 (excellent) for gastric neoplasms. The primary outcome was the visibility scale scores based on each modality and endoscope. The secondary outcome was the identification of factors including *H. pylori* infection, atrophy, location, size, morphology, histological diagnosis and intestinal metaplasia that affect the differences in visibility scale scores between TXI-1/TXI-2 and WLI.

**Results:**

Fifty-two gastric neoplasms were analyzed. The mean visibility scale scores with the NE were 2.79 ± 1.07, 3.23 ± 0.96 and 3.14 ± 0.92 for WLI, TXI-1 and TXI-2, respectively. The mean visibility scales with the CE were 2.53 ± 1.10, 3.04 ± 1.05 and 2.96 ± 1.92 for WLI, TXI-1 and TXI-2, respectively. For both endoscopes, significant differences were observed in visibility scale scores between WLI and TXI-1 (*p* < 0.001) and between WLI and TXI-2 (*p* < 0.001). The visibility scale scores of NE were superior to those of CE in all modalities. In the secondary outcome, there was no factor affected the differences of visibility scale scores between TXI-1/TXI-2 and WLI.

**Conclusions:**

This study demonstrated that TXI-1 and TXI-2 enhanced the visibility scale scores of gastric neoplasms compared with that of WLI. Moreover, newly developed endoscope has the potential to improve visibility compared to conventional endoscope.

**Trial Registration:**

This study was registered with the University Hospital Medical Information Network (UMIN000042429, 16/11/2020).

## Background

Gastric cancer was the fifth most common cancer and the third most common cause of cancer-related deaths worldwide in 2020 [1]. Surgery is the mainstay of gastric cancer treatment; however, with early detection, endoscopic treatment can be expected to provide a radical cure through minimally invasive treatment. Endoscopic screening for gastric cancer has allowed for a 30% reduction in gastric cancer mortality [2–4]. Macroscopic types of early gastric cancer (EGC) show elevation or depression, whereas the tumor color changes exhibit redness or whiteness. However, early detection of gastric cancer can be difficult, because changes in morphology and color are subtle, and endoscopists may encounter difficulty in recognizing a lesion. Moreover, EGC is highly associated with *Helicobacter pylori* (*H. pylori*) infection, and map-like redness and mucosal changes caused by *H. pylori* eradication make EGC detection difficult. Most diffuse type gastric cancers comprise an endoscopically depressed type [5]; therefore, considering these characteristics of EGC, it is important that slight changes in color and structure are detected during endoscopy.

Image-enhanced endoscopy (IEE), including narrow band imaging (NBI), blue laser imaging (BLI) and linked color imaging (LCI), was developed to improve the visibility of EGC and is currently clinically available; LCI enhances red and white hues during endoscopy and has been reported to improve gastric cancer detection and visibility [6–13]. As for the other IEEs, Yoshida et al. compared between second-generation NBI and white light imaging (WLI) in EGC detection and found no significant differences in the characteristics of the detected lesions [14]. Nagashima reported that low magnifying NBI was able to detect gastric neoplasm overlooked by WLI [15]. Dohi et al. reported that BLI-bright had a higher real-time detection rate for EGC than WLI [16].

Texture and color enhancement imaging (TXI) is a new IEE technology that has been available in clinical practice since 2020. TXI can enhance brightness, color contrast and texture changes during endoscopic observation, and has been reported to improve the visibility and color difference of gastric cancer compared with WLI [17–22]. Since the previous studies on TXI were conducted on a small number of cases or had retrospective study design, we attempted to prove the significance of TXI by a prospective case collection with sample size calculation.

This study aimed to demonstrate that TXI—which was prospectively corrected—improved the visibility of EGC compared with WLI using conventional and newly developed endoscopes. Additionally, we analyzed the effect of TXI on lesion characteristics and the improvement in visibility for EGC.

## Methods

### Patients and study design

This was a single-center, prospective trial. We prospectively enrolled patients who were diagnosed with gastric neoplasms (including adenoma and adenocarcinoma) through endoscopic and histological diagnosis, and who were referred to our hospital for treatment. Patients were enrolled as consecutive cases in this study to eliminate selection bias. Written informed consent was obtained from all patients.

The recruitment period was between August 2021 and July 2022. The exclusion criteria were as follows: age < 20 years, pregnancy, large lesions (> 8 cm) that did not fit in one endoscopic field of view, and being evaluated as inappropriate by the attending doctor for this study considering general condition. This study was approved by the Institutional Review Board of the Jikei University School of Medicine on 14 September 2020 (32–156(10,237)) and is registered with the University Hospital Medical Information Network (UMIN000042429, 16/11/2020).

### Endoscopic system and setting

We used the EVIS X1 (Olympus Corporation, Tokyo, Japan) endoscopic system and high-definition endoscopes, which include a conventional endoscope (CE) (GIF-H290Z; Olympus Corporation, Tokyo, Japan) and a newly developed endoscope (NE) (GIF-XZ1200; Olympus Corporation). Regarding the image sensor, the CE uses a Charge Coupled Device (CCD), while the NE uses a high-sensitivity Complementary Metal-Oxide-Semiconductor (CMOS) which is expected to improve image quality. The EVIS X1 system can promptly change the image modalities (WLI, TXI, and NBI) via a button on the scope holder.

### Texture and color enhancement imaging

TXI is a newly developed IEE that enhances the texture, brightness and color of endoscopic images obtained using WLI. First, the RGB input image is classified into a base and a detail layer. Second, the base layer is adjusted for brightness, followed by dynamic range compression (tone mapping). Subsequently, texture enhancement is applied to the detail layer to enhance subtle contrast. TXI mode 2 (TXI-2) is displayed by stacking the two layers, while the processing designed to expand the difference between red and white hues yields TXI mode 1 (TXI-1). TXI-1 is more tonally enhanced, while TXI-2 is more similar to WLI [23]; TXI is thought to enhance subtle morphological or color changes on the gastrointestinal surface caused by gastric neoplasms.

### Endoscopic procedure

**Fig. 1:**
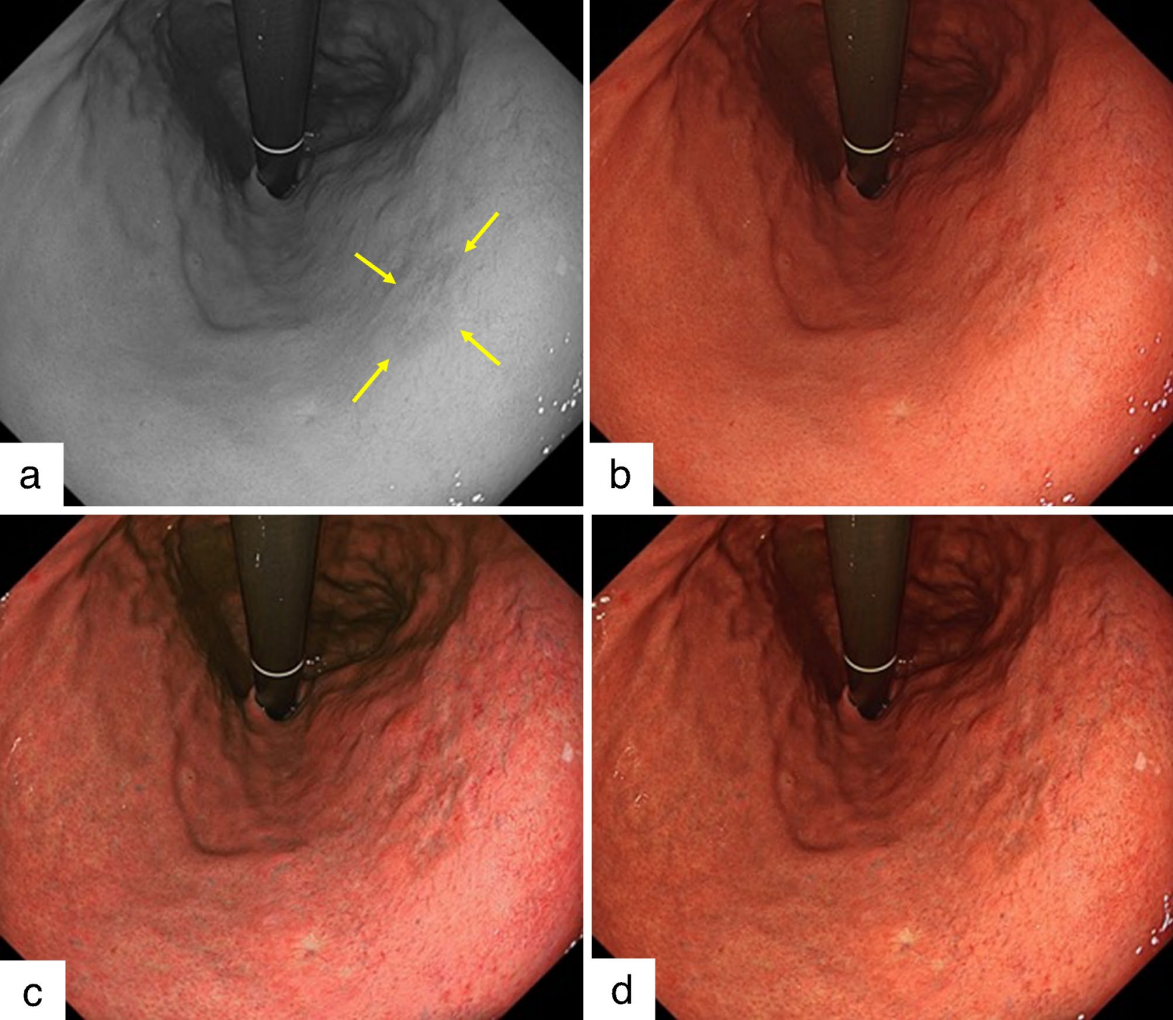
Example of early gastric cancer detected during this study. A depressed-type early gastric cancer in the lesser curvature lower body is detected using the newly developed endoscope (GIF-XZ1200, Olympus). The diagnosis of lesion margins followed the pathology finding. a. Arrows indicate lesion margins of gastric cancer in monochrome image. b. The lesion is difficult to detect in white light imaging. c. Texture and color enhancement imaging (TXI) mode 1 enhances the color and texture, and the whole image turns pinkish compared to WLI in this image. The visibility of gastric cancer is improved. d. TXI mode 2 enhances the texture, and the color tone is similar to that of WLI. The depression in the gastric cancer is enhanced

All endoscopic examinations were performed under sedation with intravenous midazolam (2–5 mg; Maruishi Pharmaceutical Co, Ltd., Osaka, Japan) or midazolam and pethidine hydrochloride (35 mg, pethidine; Takeda Pharmaceutical Company, Tokyo, Japan). Prior to treatment, an expert endoscopist performed an endoscopic examination, and the unmagnified endoscopic images of the lesion were stored in a middle-distant view with CE or NE using the three modalities (WLI, TXI-1 and TXI-2). On the day of treatment, images of the same lesion were stored in the same view as those in the other endoscope using the three modalities (Fig. 1). In total, we obtained six endoscopic images of each lesion using the three modalities and two endoscopes.

### Evaluation of endoscopic images

We created an image catalogue where each gastric neoplasm had six different images. The images were randomly arranged based on a randomized table created using Excel software (Microsoft Corporation, Redmond, Washington, USA). Six endoscopists provided the visibility scales [9, 24]. All reviewers were instructed how to apply and interpret the visibility scales by an organizer (A.D.), who was not an image reviewer in this study. Visibility scale was scored based on previous reports as follows: 1, poor (not detectable without repeated careful examination); 2, fair (hardly detectable without careful examination); 3, good (detectable with careful observation); and 4, excellent (easily detectable) [10, 25]. The reviewers comprised three expert endoscopists who were certified by the board of the Japan Gastroenterological Endoscopy Society and had experience with > 100 cases of endoscopic submucosal dissection for early gastric cancer and three novices who had experience with < 100 esophagogastroduodenoscopies.

### Outcomes

The primary outcome was the visibility scale score based on each modality and endoscope. The secondary outcome was the effect of lesion characteristics on the improvement of the visibility scale score for EGC. The status of *H. pylori* infection was defined as follows: positive (positive rapid urease test, anti-*H. pylori* antibody assay, or fecal *H. pylori* antigen assay, before eradication), eradicated (negative urease breath test or anti‐*H. pylori* antibody assay, post eradication), and negative (negative rapid urease test, anti‐*H. pylori* antibody assay, or fecal *H. pylori* antigen assay, without eradication) [26]. Atrophy was graded as open type, closed type, or negative according to the Kimura–Takemoto Classification [27]. The location of the neoplasm was defined as U (upper third), M (middle third) and L (lower third) according to the Japanese Classification of Gastric Carcinoma [28]. Morphology was classified according to the Paris classification [29], and histological diagnosis was based on Lauren’s classification [30]. A pathologist who did not know the result of visibility scale scores evaluated the degree of intestinal metaplasia classified as complete, incomplete, or negative according to the previous report [31].

### Sample size calculation

The mean visibility scale scores for EGC were reported to be 2.54 ± 1.10 (mean ± standard deviation) and 3.28 ± 0.97 for WLI and LCI, respectively [9]. TXI was expected to improve the visibility of gastric neoplasms to the same extent as that of LCI; therefore, we calculated the sample size with an α value of 0.05 and a power of 0.90 using a two-sided test. The required number of lesions was 42. Finally, considering dropout or exclusion, we set the number of cases to 50.

### Statistical analysis

All statistical analyses were performed using STATA (version 14.0; Stata Corp., College Station, Texas, United States). Quantitative parameters were compared using Student’s t test or the Mann-Whitney U test. The normal distribution was analyzed using Shapiro-Wilk test. For the secondary outcome, a two-way ANOVA was performed because the visibility scale score was evaluated repeatedly by the same reviewer. We used the visibility scale score differences between TXI-1 and WLI of NE, between TXI-2 and WLI of NE, and the following lesion characteristics for ANOVA: status of *H. pylori* infection, atrophy, location, size, morphology, histological diagnosis and intestinal metaplasia. We set the scale score differences as the dependent variables, and reviewers and each lesion characteristics as independent variables. The size was analyzed in two groups, including lesions ≥ 10 mm or < 10 mm. The significance level was set at *p* < 0.05, and Bonferroni adjustment was used when testing for repetition in ANOVA.

## Results

**Fig. 2:**
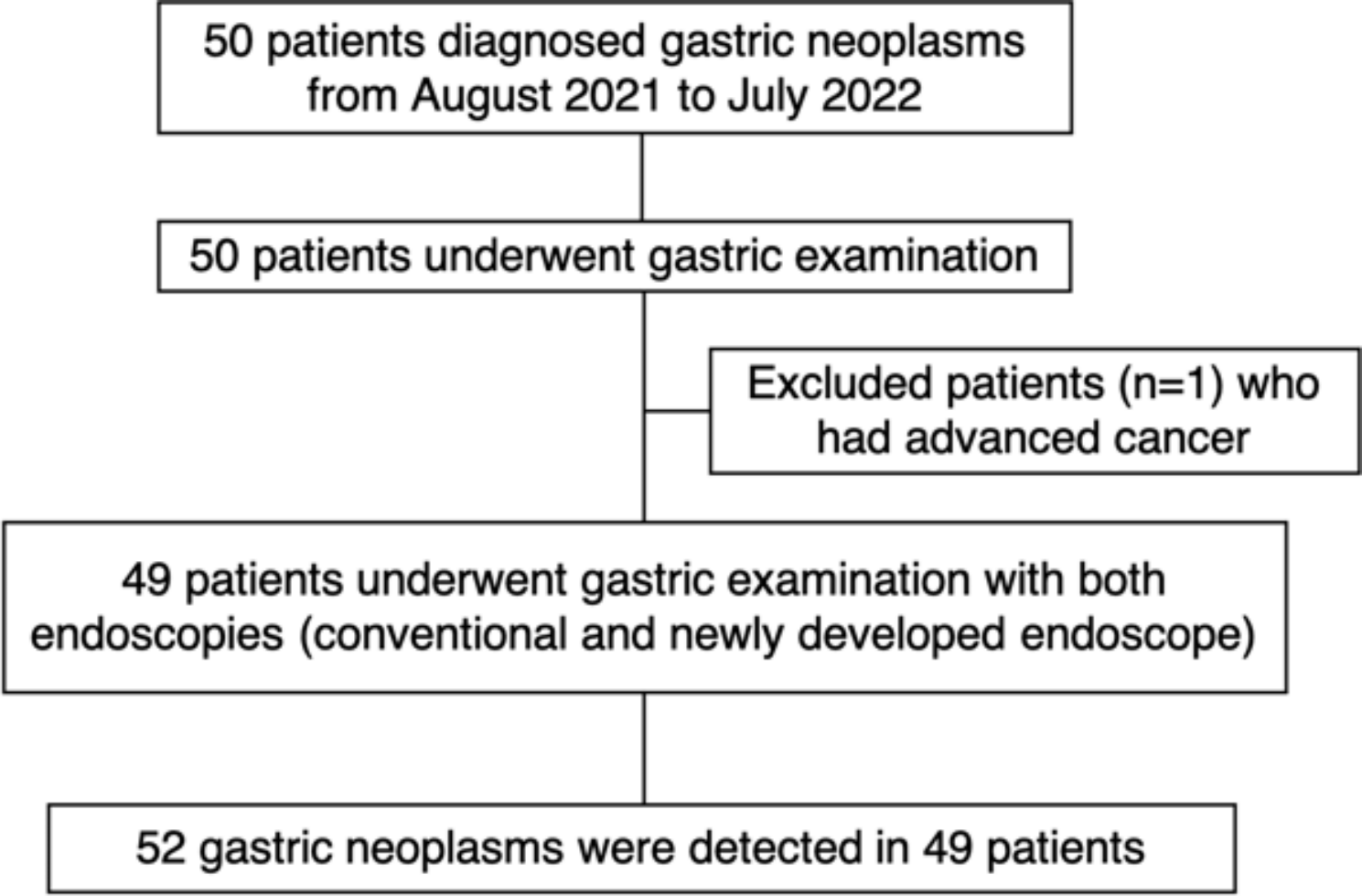
Flow chart of this study participants

Endoscopic examinations were performed in 50 patients; 1 was excluded after the first endoscopic examination because the gastric cancer was diagnosed as advanced cancer. Finally, 49 patients met the inclusion criteria, and we obtained 312 endoscopic images (6 images per lesion) from 52 lesions (Fig. 2).

Demographics of the patients and characteristics of the lesions are summarized in Table 1. The median age of the patients was 72.5 (range: 33–86) years, and 75.5% (37/49) were male. *H. pylori* infection was positive, eradicated and negative in 16, 24 and 12 lesions, respectively. Atrophy was open type, closed type and negative in 42, 7 and 3 lesions, respectively; however, all lesions without atrophy were negative for *H. pylori* infection. Regarding location, 6, 15 and 31 lesions were in U, M and L, respectively. The median (range) size of the lesions was 12.5 (1–75) mm, including 32 lesions ≥ 10 mm and 20 lesions < 10 mm. The morphology was 0-I, 0-IIa and 0-IIc in 3, 13 and 36 lesions, respectively. Histological diagnoses were diffuse type, intestinal type and adenoma in 7, 43 and 2 lesions, respectively. Intestinal metaplasia were complete, incomplete and negative in 20, 28 and 4 lesions, respectively.

**Table 1:** Demographics of the patients and characteristics of the lesions

Patients (n = 49)		
Sex	Male/Female	37/12
Age, years (range)		70.8 (33–86)
Lesions (n = 52)		
*H. Pylori* infection	Positive/Eradicated/Negative	16/24/12
Atrophy	Open type/Closed type/Negative	42/7/3
Location	U/L/M	6/15/31
Size(mm)	≥ 10, < 10	32/20
Morphology	0-I/0-IIa/0-IIb/0-IIc/0-III	3/13/0/36/0
Histological diagnosis	Diffuse type/Intestinal type/Adenoma	7/43/2
Intestinal metaplasia	Complete/Incomplete/Negative	20/28/4

U, upper third; M, middle third; L, lower third; *H. pylori*, *Helicobacter pylori*

**Fig. 3:**
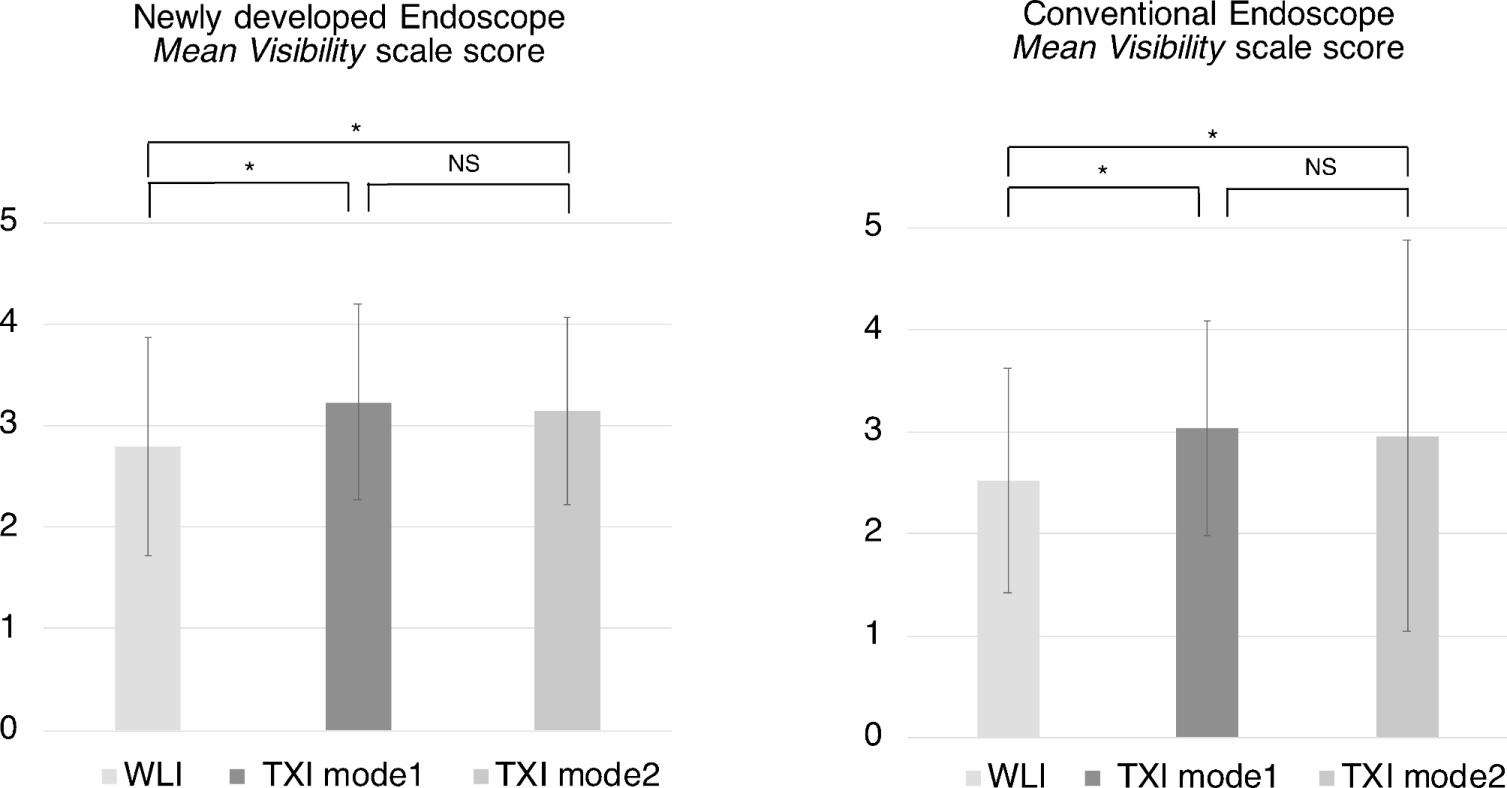
Mean visibility scale scores for the GIF-XZ1200 and GIF-H290Z endoscopes. * *p* < 0.05. WLI, white light imaging; TXI, texture and color enhancement imaging ; NS, not significant

The mean visibility scale scores based on the endoscopes and modalities are shown in Fig. 3. The mean visibility scale scores with the NE were 2.79 ± 1.07, 3.23 ± 0.96 and 3.14 ± 0.92 for WLI, TXI-1 and TXI-2, respectively. Visibility scale scores with the CE were 2.53 ± 1.10, 3.04 ± 1.05 and 2.96 ± 1.92 for WLI, TXI-1 and TXI-2, respectively. For both types of endoscopes, significant differences were observed in visibility scale scores between WLI and TXI-1 (*p* < 0.001 for both endoscopes), and WLI and TXI-2 (*p* < 0.001 for both endoscopes). When comparing the endoscopes, visibility scale scores with the NE were significantly higher than those with the CE for all modalities (WLI, *p* = 0.002; TXI-1, *p* = 0.025; and TXI-2, *p* = 0.004).

Among experts, visibility scale scores were 3.04 ± 1.00, 3.34 ± 0.90 and 3.27 ± 0.90 with the NE, and 2.89 ± 1.02, 3.15 ± 1.00 and 3.04 ± 1.02 with the CE for WLI, TXI-1 and TXI-2, respectively. Significant differences were observed in the visibility scale scores between WLI and TXI-1 (*p* = 0.008 for NE, 0.020 for CE); however, not between WLI and TXI-2 (*p* = 0.056 for NE, 0.175 for CE).

Among novices, visibility scale scores were 2.54 ± 1.07, 3.12 ± 1.01 and 3.01 ± 0.92 with the NE, and 2.16 ± 1.06, 2.92 ± 1.09 and 2.71 ± 1.11 with the CE for WLI, TXI-1 and TXI-2, respectively. For both types of endoscopes, significant differences were observed in visibility scale scores between WLI and TXI-1 (*p* < 0.001 for both endoscopes), and between WLI and TXI-2 (*p* < 0.001 for both endoscopes).

In the secondary outcome, no factors were found to significantly affect the improvement in visibility of gastric neoplasms between TXI-1 and WLI, and between TXI-2 and WLI (Table 2).

**Table 2:** Visibility scale difference between WLI and TXI-mode 1 and between WLI and TXI-mode 2 and results of two-way ANOVA in endoscopists and the six endoscopic features of gastric neoplasms

	Scale score difference (TXI mode1-WLI), mean ± SD	*p* value	Scale score difference (TXI mode2-WLI), mean ± SD	*p* value	
		TXI mode1		TXI mode2	
*H. pylori* infection		0.618		0.382	
Positive	0.34 ± 0.79		0.28 ± 0.79		
Eradicated	0.53 ± 0.94		0.44 ± 0.83		
Negative	0.38 ± 0.81		0.24 ± 0.72		
Atrophy		0.07		0.175	
Positive	0.45 ± 0.87		0.36 ± 0.81		
Open type	0.44 ± 0.86		0.34 ± 0.81		
Closed type	0.50 ± 0.93		0.48 ± 0.79		
Negative	0.22 ± 0.79		0.06 ± 0.40		
Location		0.915		0.337	
U	0.17 ± 0.69		0.08 ± 0.89		
M	0.38 ± 1.00		0.35 ± 0.78		
L	0.51 ± 0.81		0.39 0.78		
Size		0.372		0.14	
<10 mm	0.50 ± 0.89		0.48 ± 0.84		
>10 mm	0.40 ± 0.85		0.26 ± 0.76		
Morphology		0.924		0.954	
0-I	0.28 ± 0.45		0.1 ± 0.31		
0-IIa	0.55 ± 0.94		0.40 ± 0.82		
0-IIc	0.41 ± 0.86		0.34 ± 0.81		
Pathology diagnosis		0.762		0.931	
Diffuse type	0.21 ± 0.71		0.17 ± 0.48		
Intestinal type	0.47 ± 0.89		0.38 ± 0.85		
Adenoma	0.58 ± 0.64		0.25 ± 0.43		
Intestinal metaplasia		0.682		0.966	
Complete	0.30 ± 0.89		0.43 ± 0.82		
Incomplete	0.51 ± 0.88		0.34 ± 0.83		
Negative	0.56 ± 0.81		0.29 ± 0.82		

The visibility scale scores of newly developed endoscope are used for the analysis. Significance is calculated as < 0.0083 according to Bonferroni adjustment. There is no factor which affected the improvement of visibility of gastric neoplasms in TXI-1 and TXI-2.

U, upper third; M, middle third; L, lower third.

## Discussion

This study showed that TXI improved the visibility scale scores of gastric neoplasms compared with WLI using an image catalogue comprising 52 consecutive gastric neoplasms in clinical practice. Moreover, visibility scale scores with the NE were significantly better than those with CE. This study included > 50 consecutive gastric neoplasms, and selection bias was eliminated as much as possible. Visibility scale scores of TXI-1 were better than those of TXI-2; however, there was no significant difference. The encouraging outcomes from this benchmark test suggest that TXI may enhance lesion visibility, regardless of the prevailing conditions. A randomized controlled trial is warranted to evaluate whether TXI can enhance the detection or delineation of EGC.

Recently, LCI was developed as a new IEE to assist in detecting gastrointestinal neoplasms. LCI enhances the color change in endoscopic images obtained using WLI. Some reports have shown that LCI enhances the visibility of gastric neoplasms, and three randomized controlled trials revealed that LCI improved the detection rate of upper gastrointestinal neoplasms [11, 13, 32]. As expected, it was demonstrated that focusing on color change was effective for detecting upper gastrointestinal neoplasms during endoscopy. Although LCI enhances the brightness and visibility of red and white hues, TXI-1 and TXI-2 can enhance the morphological changes in the gastrointestinal mucosa. Considering that the mean visibility scale scores in TXI-2 were significantly higher than those in WLI, endoscopists should monitor the change in elevation/depression to detect EGC.

TXI-1 had the highest visibility scale scores compared to those of TXI-2 and WLI. These results may be owing to several reasons. First, completely flat type (0-IIb) EGC is rare [29, 33], and no lesions were classified as 0-IIb in this study. Second, some EGCs do not exhibit color changes, and it may be difficult to identify the lesion despite color enhancement in the endoscopic image. Therefore, it would be reasonable to improve the visibility of EGC by enhancing both the texture and color, as in TXI-1. However, it remains unknown whether color or morphological changes are more effective in improving the visibility of EGC.

An analysis of color changes between the inside and outside of a lesion with L* a* b* values was used to objectively evaluate visibility [34]. Using L* a* b* values, Abe et al. [17] reported a significant color difference between the inside and outside of lesions for WLI and TXI (both TXI-1 and − 2), whereas Ishikawa et al. [18] reported a significant color difference between WLI and TXI-1. Moreover, Koyama et al. [19] also reported that color difference between EGCs and non-neoplastic mucosa was significantly higher in TXI than in WLI in all patients. We did not analyze the color difference using L* a* b* values since a similar result was likely to be obtained in our study. As reported in our previous clinical study using esophageal neoplasms, the L* a* b* values and visibility scale scores may not completely correspond to one another [35]. In addition to color differences, other information—such as morphological changes and mucosal or microvascular patterns—may affect the visibility of EGC.

We analyzed characteristics whereby TXI significantly improved visibility scale scores of EGC by considering the impact among endoscopists who evaluated the endoscopic images; however, we could not extract the characteristics. The results showed a tendency for TXI to always be superior to WLI, regardless of the lesion characteristics or endoscopist’s experience. Particularly, TXI can enhance the visibility of EGC regardless of lesion color, morphological type, location, the status of *H. pylori* infection, atrophic gastritis, and histology and intestinal metaplasia. This result was similar to that previously reported in studies examining the visibility of gastric neoplasms using LCI [9, 10].

In this study, TXI resulted in better gastric neoplasms visibility scale scores than WLI; however, it remains unclear whether TXI actually improved EGC detection. The effectiveness of NBI in the detection of EGC is still controversial. The usefulness of LCI in detecting neoplasm in the upper gastrointestinal tract has been reported in a randomized controlled trials, and further studies should be conducted to determine which IEE is most effective. The improved resolution may also contribute to the detection rates, because this study demonstrates that the visibility scale scores of NE was significantly better than that of CE. Although 0-IIb lesions with minimal color changes are rare, TXI has limited visibility enhancement for these lesions. Thus, other modalities, such as magnifying endoscopy and NBI, may be superior for detecting 0-IIb lesions [36].

This study had some limitations. First, the modalities were not completely blinded while scoring the visibility scale. Therefore, it is undeniable that reviewers may have rated TXI higher than WLI. Second, endoscopic examinations were conducted by expert endoscopists at a single center. Since it is necessary to maintain high-quality examinations and obtain appropriate endoscopic images, we included endoscopists with experience in the protocol. Third, the images captured with each endoscope and modality differed; six images were acquired per lesion at different times, and the conditions varied slightly depending on gastric peristalsis, air insufflation and endoscope stability.

## Conclusions

TXI improved the visibility scale scores of gastric neoplasms compared with those of WLI. Moreover, NE has the potential to improve visibility compared to CE.

## Data Availability

The datasets used and/or analyzed during the current study are available from the corresponding author on reasonable request.

## Abbreviations

ANOVA: Analysis of variance
CE: Conventional endoscope
EGC: Early gastric cancer
IEE: Image-enhanced endoscopy
LCI: Linked color imaging
NBI: Narrow-band imaging
NE: Newly developed endoscope
TXI: Texture and color enhancement imaging
WLI: White light imaging

## References

[1] Ferlay J, Colombet M, Soerjomataram I, Parkin DM, Pineros M, Znaor A et al. Cancer statistics for the year 2020: an overview. Int J Cancer. 2021. 10.1002/ijc.33588 33818764

[2] Machlowska J, Baj J, Sitarz M, Maciejewski R, Sitarz R (2020). Gastric cancer: epidemiology, risk factors, classification, genomic characteristics and treatment strategies. Int J Mol Sci.

[3] Matsumoto S, Ishikawa S, Yoshida Y (2013). Reduction of gastric cancer mortality by endoscopic and radiographic screening in an isolated island: a retrospective cohort study. Aust J Rural Health.

[4] Hamashima C, Ogoshi K, Okamoto M, Shabana M, Kishimoto T, Fukao A (2013). A community-based, case-control study evaluating mortality reduction from gastric cancer by endoscopic screening in Japan. PLoS ONE.

[5] Kim GH (2021). Systematic endoscopic approach to early gastric cancer in clinical practice. Gut Liver.

[6] Yoshifuku Y, Sanomura Y, Oka S, Kurihara M, Mizumoto T, Miwata T (2017). Evaluation of the visibility of early gastric cancer using linked color imaging and blue laser imaging. BMC Gastroenterol.

[7] Kanzaki H, Takenaka R, Kawahara Y, Kawai D, Obayashi Y, Baba Y (2017). Linked color imaging (LCI), a novel image-enhanced endoscopy technology, emphasizes the color of early gastric cancer. Endosc Int Open.

[8] Fukuda H, Miura Y, Osawa H, Takezawa T, Ino Y, Okada M (2019). Linked color imaging can enhance recognition of early gastric cancer by high color contrast to surrounding gastric intestinal metaplasia. J Gastroenterol.

[9] Kitagawa Y, Suzuki T, Hara T, Nankinzan R, Takashiro H, Sugita O (2019). Linked color imaging improves the endoscopic visibility of gastric mucosal cancers. Endosc Int Open.

[10] Khurelbaatar T, Miura Y, Osawa H, Nomoto Y, Tokoro S, Tsunoda M (2022). Usefulness of linked color imaging for the detection of obscure early gastric cancer: multivariate analysis of 508 lesions. Dig Endosc.

[11] Gao J, Zhang X, Meng Q, Jin H, Zhu Z, Wang Z (2021). Linked color imaging can improve detection rate of early gastric cancer in a high-risk population: a multi-center randomized controlled clinical trial. Dig Dis Sci.

[12] Ono H, Yao K, Fujishiro M, Oda I, Uedo N, Nimura S (2021). Guidelines for endoscopic submucosal dissection and endoscopic mucosal resection for early gastric cancer (second edition). Dig Endosc.

[13] Wu CCH, Namasivayam V, Li JW, Khor CJ, Fock KM, Law NM (2021). A prospective randomized tandem gastroscopy pilot study of linked color imaging versus white light imaging for detection of upper gastrointestinal lesions. J Gastroenterol Hepatol.

[14] Yoshida N, Doyama H, Yano T (2021). Early gastric cancer detection in high-risk patients: a multicentre randomised controlled trial on the effect of second-generation narrow band imaging. Gut.

[15] Nagashima R (2022). Low-magnification narrow-band imaging for small gastric Neoplasm detection on screening endoscopy. VideoGIE.

[16] Dohi O, Yagi N, Naito Y (2019). Blue laser imaging-bright improves the real-time detection rate of early gastric cancer: a randomized controlled study. Gastrointest Endosc.

[17] Abe S, Yamazaki T, Hisada IT, Makiguchi ME, Yoshinaga S, Sato T (2022). Visibility of early gastric cancer in texture and color enhancement imaging. DEN Open.

[18] Ishikawa T, Matsumura T, Okimoto K, Nagashima A, Shiratori W, Kaneko T (2021). Efficacy of texture and color enhancement imaging in visualizing gastric mucosal atrophy and gastric Neoplasms. Sci Rep.

[19] Koyama Y, Sugimoto M, Kawai T, Mizumachi M, Yamanishi F, Matsumoto S (2023). Visibility of early gastric cancers by texture and color enhancement imaging using a high-definition ultrathin transnasal endoscope. Sci Rep.

[20] Kawasaki A, Yoshida N, Nakanishi H, Tsuji S, Takemura K, Doyama H (2023). Usefulness of third-generation narrow band imaging and texture and color enhancement imaging in improving visibility of superficial early gastric cancer: a study using color difference. DEN Open.

[21] Kemmoto Y, Ozawa SI, Sueki R (2024). Higher detectability of gastric cancer after Helicobacter pylori eradication in texture and color enhancement imaging mode 2 in screening endoscopy. DEN Open.

[22] Shijimaya T, Tahara T, Uragami T (2023). Usefulness of texture and color enhancement imaging (TXI) in early gastric cancer found after Helicobacter pylori eradication. Sci Rep.

[23] Sato T (2021). TXI: texture and color enhancement imaging for endoscopic image enhancement. J Healthc Eng.

[24] Yoshida N, Hisabe T, Hirose R, Ogiso K, Inada Y, Konishi H (2015). Improvement in the visibility of colorectal polyps by using blue laser imaging (with video). Gastrointest Endosc.

[25] Kitagawa Y, Suzuki T, Nankinzan R, Ishigaki A, Furukawa K, Sugita O (2020). Comparison of endoscopic visibility and miss rate for early gastric cancers after Helicobacter pylori eradication with white-light imaging versus linked color imaging. Dig Endosc.

[26] Kato M, Ota H, Okuda M, Kikuchi S, Satoh K, Shimoyama T (2019). Guidelines for the management of Helicobacter pylori Infection in Japan: 2016 revised Edition. Helicobacter.

[27] Kimura K, Takemoto T (1969). An endoscopic recognition of the atrophic border and its significance in chronic gastritis. Endoscopy.

[28] Japanese Gastric Cancer Association (2017). Japanese gastric cancer treatment guidelines 2014 (ver. 4). Gastric Cancer.

[29] The Paris endoscopic (2003). Classification of superficial neoplastic lesions: esophagus, stomach, and colon: November 30 to December 1, 2002. Gastrointest Endosc.

[30] Lauren P, THE TWO HISTOLOGICAL MAIN TYPES OF GASTRIC CARCINOMA (1965). DIFFUSE AND SO-CALLED INTESTINAL-TYPE CARCINOMA. AN ATTEMPT AT A HISTO-CLINICAL CLASSIFICATION. Acta Pathol Microbiol Scand.

[31] Kanemitsu T, Uedo N, Ono T (2023). Magnifying endoscopy with narrow-band imaging for diagnosis of subtype of gastric intestinal metaplasia. J Gastroenterol Hepatol.

[32] Ono S, Kawada K, Dohi O, Kitamura S, Koike T, Hori S (2021). Linked color imaging focused on Neoplasm detection in the upper gastrointestinal tract: a randomized trial. Ann Intern Med.

[33] Endoscopic Classification Review Group (2005). Update on the Paris classification of superficial neoplastic lesions in the digestive tract. Endoscopy.

[34] Kuehni RG (1976). Color-tolerance data and the tentative CIE 1976 L a b formula. J Opt Soc Am.

[35] Dobashi A, Ono S, Furuhashi H, Futakuchi T, Tamai N, Yamauchi T (2021). Texture and color enhancement imaging increases color changes and improves visibility for squamous cell carcinoma suspicious lesions in the pharynx and esophagus. Diagnostics (Basel).

[36] Eleftheriadis N, Inoue H, Ikeda H, Maselli R, Onimaru M, Yoshida A (2014). Improved optical identification of laterally spreading type 0-IIb gastric lesion with narrow band imaging magnification endoscopy. Ann Gastroenterol.

